# Evaluation of the effectiveness of surgical resection and ablation for the treatment of early‐stage hepatocellular carcinoma: A retrospective cohort study

**DOI:** 10.1002/cnr2.2030

**Published:** 2024-03-15

**Authors:** Bilan Yang, Xiaoli Xi, Hongsheng Yu, Hao Jiang, Zixi Liang, Abdukyamu Smayi, Bin Wu, Yidong Yang

**Affiliations:** ^1^ Department of Gastroenterology The Third Affiliated Hospital of Sun Yat‐Sen University Guangzhou People's Republic of China; ^2^ Guangdong Provincial Key Laboratory of Liver Disease Research Guangzhou Guangdong People's Republic of China

**Keywords:** cancer risk factors, liver cancer, surgical therapy, survival

## Abstract

**Background:**

The optimal treatment strategy for early‐stage hepatocellular carcinoma (HCC) remains controversial, specifically in regard to surgical resection (SR) and ablation. The aim of this study was to investigate the impact of SR and ablation on recurrence and prognosis in early‐stage HCC patients, to optimize treatment strategies and improve long‐term survival.

**Methods:**

A retrospective analysis was conducted on 801 patients diagnosed with Barcelona Clinic Liver Cancer (BCLC) stage 0/A HCC and treated with SR or ablation between January 2015 and December 2019. The effectiveness and complications of both treatments were analyzed, and patients were followed up to measure recurrence and survival. Propensity score matching (PSM) was employed to increase comparability between the two groups. The Kaplan–Meier method was used to analyze recurrence and survival, and a Cox risk proportional hazard model was used to identify risk factors that affect recurrence and surviva.

**Results:**

Before PSM, the overall survival (OS) rates were similar in both groups, with recurrence‐free survival (RFS) rates better in the SR group than in the ablation group. After PSM, there was no significant difference in OS between the two groups. However, the RFS rates were significantly better in the SR group than in the ablation group. The ablation group exhibited superior outcomes compared to the SR group, with shorter treatment times, reduced bleeding, shorter hospital stays, and lower hospital costs. Concerning the location of the HCC within the liver, comparable efficacy was observed between SR and ablation for disease located in the noncentral region or left lobe. However, for HCCs located in the central region or right lobe of the liver, SR was more effective than ablation.

**Conclusions:**

This study revealed no significant difference in OS between SR and ablation for early‐stage HCC, with SR providing better RFS and ablation demonstrating better safety profiles and lower hospital costs. These findings offer valuable insights for clinicians in determining optimal treatment strategies for early‐stage HCC patients, particularly in terms of balancing efficacy, safety, and cost considerations.

## 1 INTRODUCTION

In 2020, primary liver cancer accounted for approximately 906 000 new cases worldwide, making it the sixth most prevalent malignancy. Approximately 830 000 of these patients died, ranking liver cancer as the third leading cause of cancer‐related mortality.^1^ Hepatocellular carcinoma represents the most prevalent subtype (75–85%) of liver cancer.^2^, ^3^ Effective management of early‐stage tumors is a critical challenge in the treatment of HCC, as it plays a vital role in improving overall survival. The early detection rates of HCC are increasing in Asian endemic countries, attributable to the importance of HCC surveillance in high‐risk populations and continuous advancements in liver cancer screening tests and technologies.^4^

Radical treatment remains the cornerstone for early‐stage HCC. The 2022 update of the Barcelona staging system endorsed hepatectomy, ablation, and liver transplantation as radical treatment modalities for very early and early‐stage (Barcelona Clinic Liver Cancer (BCLC) 0/A) HCC.^5^ Guidelines from the European Association for the Study of the Liver (EASL), the Asian Pacific Association for the Study of the Liver (APASL), and the Association for the Study of Liver Diseases (AASLD) concur on these recommendations.^6^, ^7^, ^8^ Nevertheless, challenges such as the scarcity of donor livers, the intricate nature of liver transplantation, a notable incidence of postoperative complications, and elevated costs have impeded the widespread adoption of liver transplantation. Consequently, surgical resection (SR) and ablation have become the most frequently used treatments for early‐stage HCC.

Several previous studies have yielded comparable outcomes between SRs and local ablation as treatment options for early‐stage HCC.^9^, ^10^, ^11^, ^12^, ^13^, ^14^, ^15^ However, recent studies have presented conflicting findings, with SRs outperforming radiofrequency ablation (RFA) in terms of both local recurrence rates and long‐term outcomes.^16^, ^17^, ^18^, ^19^, ^20^, ^21^, ^22^, ^23^, ^24^ Most prior RCTs and retrospective studies have suffered from small sample sizes and inadequate follow‐up. Additionally, the majority of studies have concentrated on comparing the outcomes of SR and ablation for HCCs less than 3 cm, with fewer investigations comparing the two treatments for tumors exceeding 3 cm and for HCCs located in different regions of the liver.

Hence, we conducted a retrospective study that included subgroup analyses of patients with HCC up to 5 cm in length and patients with different tumor sites (central or peripheral HCC, left HCC, or right HCC) to comprehensively compare the long‐term survival and recurrence rates associated with resection and ablation. To mitigate the influence of confounding factors, propensity score matching (PSM) was used to analyze the baseline characteristics of the patients in the study cohorts.

## 2 MATERIALS AND METHODS

### 2.1 Patients

**FIGURE 1:**
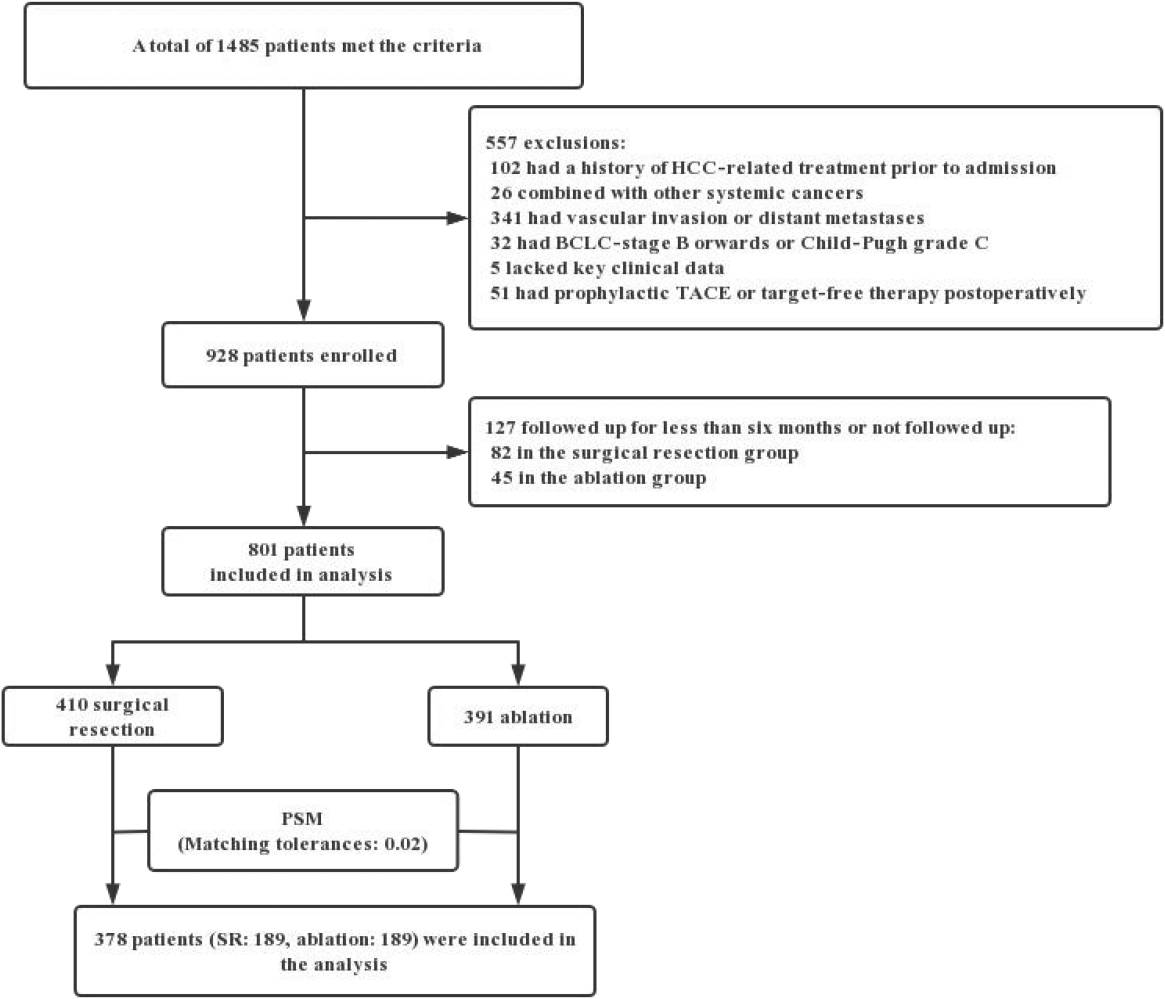
Flow chart of the study design.

A retrospective analysis was conducted on 801 patients diagnosed with BCLC stage 0/A HCC at a single hospital between January 2015 and December 2019. Patients who underwent either SR or ablation were included in the study if they met certain criteria: (1) ≥18 years of age; (2) initial HCC diagnosis confirmed by pathological evidence or EASL guidelines (2018 edition),^6^ with diagnosis in the absence of biopsy evidence primarily based on ultrasound or enhanced spiral computed tomography (CT) or magnetic resonance imaging (MRI); (3) liver function Child–Pugh grade A/B; and (4) no visible portal/hepatic vein invasion or distant metastases. The exclusion criteria included a history of previous HCC treatment, such as surgery, ablation, transarterial chemoembolization, radiotherapy, chemotherapy, immunotherapy, or targeted drug therapy; a history of other malignancies in the last 5 years; no postoperative follow‐up or follow‐up of less than 6 months; and missing key information, such as clinical and laboratory data. The complete screening procedure is detailed in Figure 1. Baseline patient information, including demographic data, potential causes of HCC, laboratory test data, and tumor‐related imaging data, was collected. Treatment‐related information, such as perioperative conditions, complications, length of hospital stay, and costs, was recorded. Follow‐up was conducted via inpatient/outpatient information every 3–6 months for the first 2 years after surgery and every 6–12 months thereafter until the end of the study. Postoperative recurrence, follow‐up treatment, and HCC survival were recorded. The end of follow‐up was December 31, 2022, and the primary endpoint event was overall survival, with recurrence rate and recurrence‐free survival as secondary endpoint events. The study was approved by the Ethics Committee of the Third Affiliated Hospital of Sun Yat‐sen University.

### 2.2 Diagnosis and definition

The present study validated the diagnosis of hepatocellular carcinoma through histopathology analysis in subjects subjected to surgical resection and through pathology or diagnostic criteria in accordance with the 2018 edition of the EASL guidelines^6^ in subjects subjected to ablation. For the purposes of this investigation, early‐stage HCC was defined as encompassing BCLC stage 0/A. Specifically, BCLC stage 0 is characterized by a solitary tumor of ≤2 cm in diameter, while BCLC stage A entails a solitary or multiple tumors each of ≤3 cm in diameter, according to the 2022 edition of the Barcelona guidelines.

### 2.3 Statistics

The present study utilized SPSS 25.0 and GraphPad Prism 7.0 for performing the statistical analyses. PSM was employed to mitigate the impacts of confounding and selection bias, with known or assumed confounders being used as dependent variables (such as age, sex, viral infections, cirrhosis, family history, combined chronic diseases, BMI, WBC, HGB, PLT, AST, ALB, TB, AFP, Child–Pugh classification, number of tumors, and tumor diameter) to match enrolled patients using the 1:1 nearest neighbor matching method (matching tolerance of 0.02). Continuous variables that met the normal distribution assumption were subjected to *t* tests, while those that did not conform were subjected to Mann–Whitney U tests. Categorical variables are represented as composition ratios and were subjected to either Chi‐square or Fisher's exact tests to determine differences. Overall survival (OS) and recurrence‐free survival (RFS) were compared between the two groups using Kaplan–Meier and log‐rank tests, and the prognostic importance of each variable for predicting OS and RFS was assessed via univariate and multivariate Cox proportional hazards regression models. Variables with *p* < .05 according to the univariate analysis were included in the multivariate analysis, and the results are reported as risk ratios (HRs) with 95% confidence intervals (CIs). A *p* value <.05 was considered indicative of statistical significance.

## 3 RESULTS

### 3.1 General clinical information

This study analyzed the demographic and clinical characteristics of 801 patients with HCC; the majority were male (88.1%) and infected with hepatitis B virus (*n* = 738, 92.1%) (Figure S1). Patients in the ablation group were older (median age 52 vs. 53 years, *p* = .046), had higher rates of hepatitis C virus infection (0.7% vs. 4.1%, *p* < .001), elevated bilirubin levels (12.90 vs. 14.05, *p* < .001) and AST levels (31 vs. 34, *p* = .01), and lower leukocyte counts (5.7 vs. 5.1, *p* < .001), platelet counts (161 vs. 133, *p* < .001), and albumin levels(41.3 vs. 40.3, *p* = .02). Moreover, the ablation group exhibited smaller tumor diameters (32 vs. 20 mm, *p* < .001). Conversely, the SR group demonstrated a greater proportion of single tumors (96.8% vs. 85.4%, *p* < .001) and a greater number of patients classified as Child–Pugh grade A (92.0% vs. 81.1%, *p* < .001) than the ablation group (Table 1). To ensure comparability between the two groups, PSM was performed to balance the distribution of baseline variables. Ultimately, 189 patients were included in each group following PSM. The results indicated no significant differences in baseline information between the two groups (*p* > .05) (Table S1).

**TABLE 1:** General clinical information of patients in SR and ablation (total cohort).

Variables	SR (*n* = 410)	Ablation (*n* = 391)	*p* value
Age (Year)	51.8 ± 11.3	53.4 ± 11.1	*p* = .046
Sex (*n*,%)			*p* = .190
Male	355 (86.6)	351(89.8)	
Female	55 (13.4)	40(10.2)	
Viral infections (*n*,%)			
HBV	384 (93.7)	356 (91.0)	*p* < .001
HCV	3 (0.7)	16 (4.1)	*p* < .001
Cirrhosis (*n*,%)	270 (65.9)	265 (67.8)	*p* = .600
Family History (*n*,%)	25 (6.1)	35 (9.0)	*p* = .140
Combine chronic diseases (*n*,%)	88 (21.5)	80 (20.5)	*p* = .729
BMI	23.1 ± 3.3	22.8 ± 3.4	*p* = .231
WBC (×10^9^/L)	5.7 (2.4)	5.1 (2.2)	*p* < .001
Hb (g/L)	142 (20)	141 (23)	*p* = .149
PLT (×10^9^/L)	166 (97)	131 (89)	*p* < .001
AST (U/L)	31 (19)	34 (28)	*p* = .010
ALT (U/L)	32 (25)	35 (25)	*p* = .158
ALB (g/L)	41.30 (5.5)	40.30 (6.8)	*p* = .020
TBil (umol/L)	12.90 (7.7)	14.05 (11.0)	*p* < .001
AFP (ng/ml)	35.64 (337.5)	23.86 (183.8)	*p* = .165
Child‐Pugh classification (*n*,%)			*p* < .001
A	377 (92.0)	317 (81.1)	
B	33 (8.0)	74 (18.9)	
Number of tumors (*n*,%)			*p* < .001
Single	397 (96.8)	334 (85.4)	
Multiple	13 (3.2)	57 (14.6)	
Tumor diameter (mm)	32 (21)	20 (11)	*p* < .001

Abbreviations: AFP, alpha‐fetoprotein; ALB, albumin; ALT, alanine aminotransferase; AST, aspartate transaminase; HBV, hepatitis B virus; HCV, hepatitis C virus; BMI, Body Mass Index; Hb, hemoglobin; PLT, platelet; SR, surgical resection; TBil, total bilirubin.

### 3.2 Ablation therapy produced superior short‐term treatment outcomes

Following PSM, the comparative analysis of perioperative outcomes between the ablation group and the SR group revealed notable differences in several perioperative parameters. Specifically, compared with the ablation group, the SR group exhibited significantly greater blood loss (100 vs. 0 mL, *p* < .001), a significantly greater intraoperative transfusion rate (21.2% vs. 9.0%, *p* < .001), and greater postoperative analgesia requirements (86.2% vs. 3.7%, *p* < .001). Conversely, the ablation group demonstrated a significantly shorter median operative time (3.0 vs. 1.5 h, *p* < .001) and median hospital stay (17 days vs. 14 days, *p* < .001) and lower hospital costs (57347.1 yuan vs. 42, 100.6 yuan, *p* < .001) than the SR group (Table 2). Postoperative complications were assessed utilizing the Dindo‐Demartines‐Clavien classification.^25^ Notably, the incidence of Grade II or higher complications was significantly greater in the SR group than in the ablation group (14.8% vs. 6.3%, *p* = .011) (Table S2). In the SR group, the primary complications included lung infection (6/189), intra‐abdominal hemorrhage (1/189), poor incision healing (3/189), bile leakage (4/189), pleural effusion (3/189), peritoneal effusion (4/189), liver failure (2/189), acute intestinal obstruction (1/189), peritonitis (1/189), acute respiratory failure (2/189), and acute heart failure (1/189). On the other hand, in the ablation group, the main complications were lung infection (5/189), intra‐abdominal hemorrhage (1/189), pleural effusion (3/189), peritonitis (2/189), and acute intestinal obstruction (1/189).

**TABLE 2:** Perioperative information for patients in SR and ablation (after PSM).

Variables	SR *n* = 189	Ablation *n* = 189	*p* value
Operating time (h)	3.00 (1.50)	1.50 (1.00)	*p* < .001
Blood loss (ml)	100 (100)	0 (5)	*p* < .001
Intraoperative blood transfusion (*n*,%)			*p* < .001
Yes	40 (21.2)	17 (9.0)	
No	149 (78.8)	172 (91.0)	
Postoperative analgesia (*n*,%)			*p* < .001
Yes	163 (86.20	7 (3.7)	
No	26 (13.8)	182 (96.3)	
Length of hospital stay (*d*)	17 (9)	14 (9)	*p* < .001
Total hospitalization costs ($)	57347.1 (25803.5)	42100.6 (14857.0)	*p* < .001

Abbreviations: PSM, propensity score matching; SR, surgical resection.

### 3.3 Comparable overall survival rates were observed in patients who underwent surgical resection and ablation treatment

**FIGURE 2:**
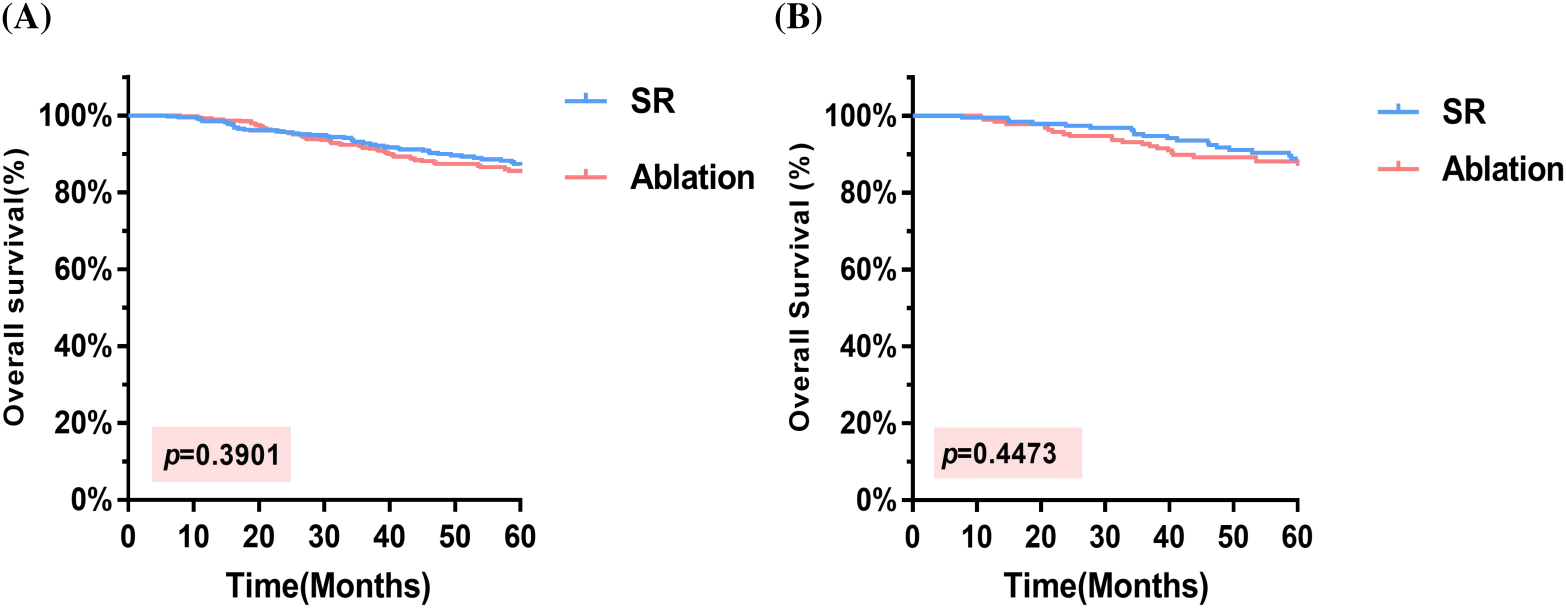
K–M curves comparing OS. (A) In the total cohort, the 5‐year OS rate was 88.2% in the SR group and 86.7% in the ablation group. (B) After PSM, the 5‐year OS rates were observed to be 89.9% and 88.3%, respectively. OS, overall survival; PSM, propensity score matching; SR, surgical resection.

Prior to PSM, the cohort exhibited a median follow‐up time of 61.2 months, and the median overall survival was indeterminate in both groups. Throughout the follow‐up period, a total of 100 patients died, resulting in overall mortality rates of 11.7% (48/410) and 13.2% (52/391) in the respective groups. The OS rates at 1, 3, and 5 years were 98.5%, 92.9%, and 88.2%, respectively, in the SR group and 99.2%, 91.5%, and 86.7%, respectively, in the ablation group; moreover, no notable difference in OS rates was observed between the groups (HR = 0.8422, 95% CI = 0.5689–1.247; *p* = .3901) (Figure 2a). After PSM, the median follow‐up time was 61.5 months. The mortality rates in the SR and ablation groups were 10.1% (19/189) and 11.6% (22/189), respectively. The OS rates at 1, 3, and 5 years were 99.4%, 94.7%, and 89.9%, respectively, in the SR group and 98.9%, 92.5%, and 88.3%, respectively, in the ablation group, with no substantial difference in OS rates detected between the groups (HR = 0.789, 95% CI = 0.4275–1.456; *p* = .4473) (Figure 2b).

### 3.4 RFS was greater in patients who underwent surgical resection than in those who underwent ablation

**FIGURE 3:**
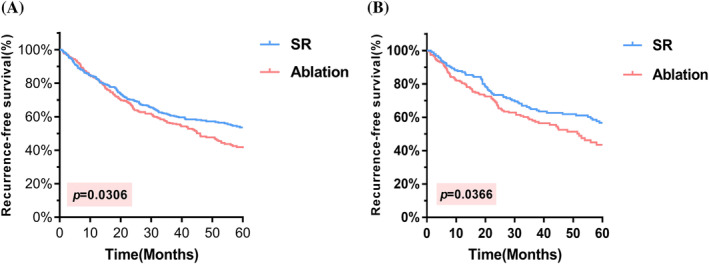
K–M curves comparing RFS. (A) In the total cohort, the 5‐year RFS rates were 60.0% for SR and 51.4% for ablation, respectively. (B) After PSM, the 5‐year RFS rates were 62.9% for SR and 54.0% for ablation. PSM, propensity score matching; RFS, recurrence‐free survival; SR, surgical resection.

The median follow‐up duration was 53.5 months prior to PSM. In the SR group, the median RFS was not reached, while in the ablation group, the median RFS was 46 months. The overall recurrence rates were 40.1% (164/410) and 48.5% (190/391) in the two respective groups. The 1‐year, 3‐year, and 5‐year RFS rates were 82.7%, 64.4%, and 60.0%, respectively, in the SR group and 82.6%, 59.1%, and 51.4%, respectively, in the ablation group. Notably, the SR group exhibited a superior RFS than the ablation group (HR = 0.795, 95% CI = 0.6453–0.9793, *p* = .0306) (Figure 3a). After PSM, the median follow‐up duration was 58.3 months, and the median RFS was not achieved in the SR group, whereas the ablation group exhibited a median RFS of 52 months. The overall recurrence rates were 37.0% (70/189) and 46.0% (87/189), respectively. The 1‐year, 3‐year, and 5‐year RFS rates were 87.3%, 68.2%, and 62.9%, respectively, in the SR group and 80.4%, 60.8%, and 54.0%, respectively, in the ablation group. Importantly, patients who underwent SR had significantly better RFS than did those who underwent ablation (HR = 0.717, 95% CI = 0.5241–0.9809, *p* = .0366) (Figure 3b). The primary sites of tumor recurrence included intrahepatic, extrahepatic, bone, and lymph nodes, among others. For patients encountering tumor recurrence, diverse treatment modalities were utilized, comprising SR, local ablation, transcatheter arterial chemoembolization (TACE), radiotherapy, targeted therapy, and immunotherapy (Table S3). Concerning the optimal treatment and prognosis following recurrence, follow‐up continues with the aim of gathering extended‐term survival information.

### 3.5 RFS superior in surgical resection compared to ablation for single tumor diameter 3–5 cm

**FIGURE 4:**
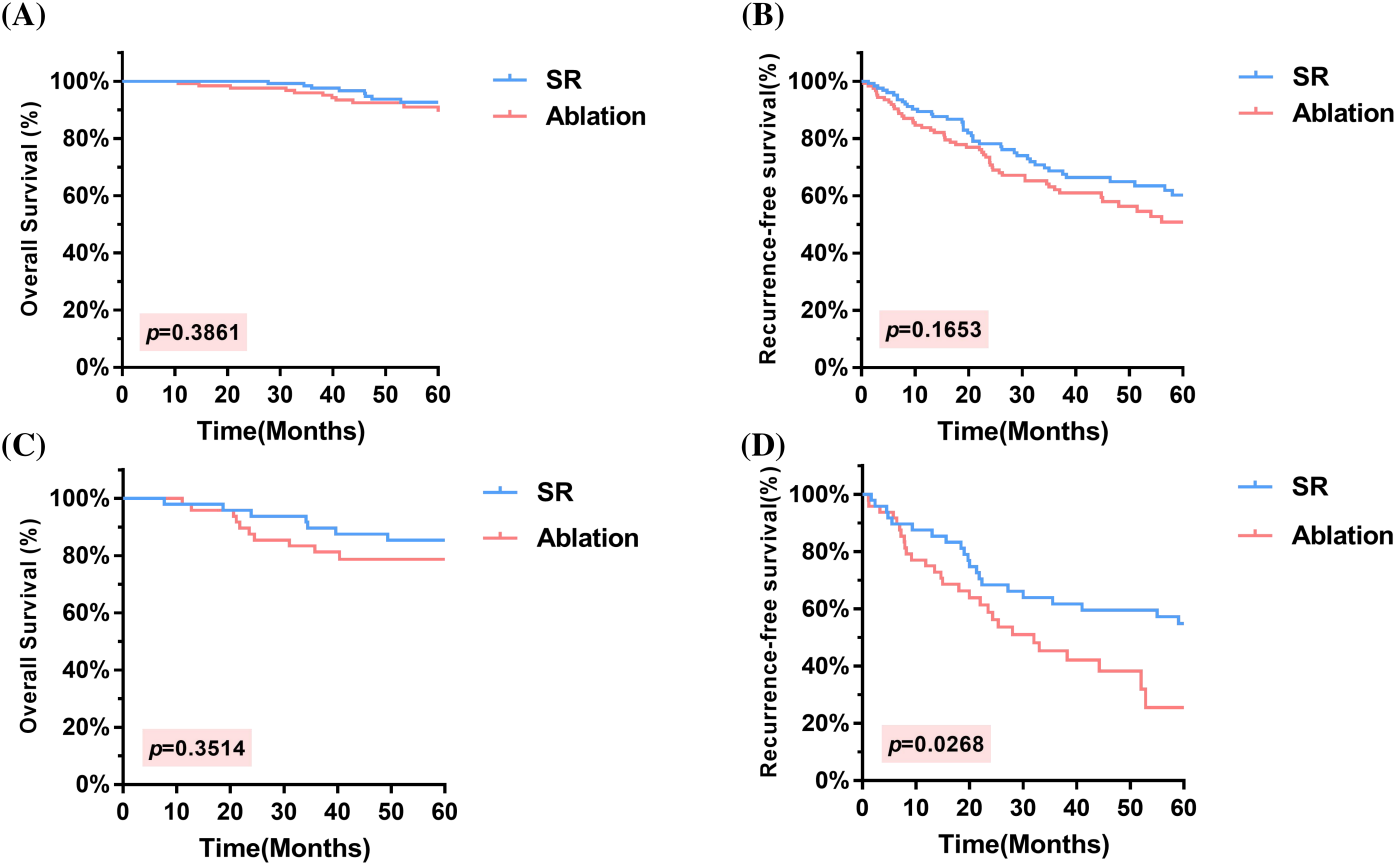
K–M curves comparing OS and RFS in different subgroups. (A, B) According to the subgroup analysis of patients with a solitary HCC lesion<3 cm, there were no significant differences in OS or RFS. (C, D) for patients with solitary HCC 3–5 cm, while the difference in OS between the two groups was not statistically significant, the RFS was better in the SR group than in the ablation group. HCC, hepatocellular carcinoma; OS, overall survival; RFS, recurrence‐free survival; SR, surgical resection.

In the present study, we aimed to investigate the impact of SR and ablation treatments on OS and RFS in patients with single‐tumor diameters <3 cm and 3–5 cm. The results indicated no significant difference in OS (HR = 0.6705, 95% CI = 0.2726–1.649, *p* = .3861) and RFS (HR = 0.7471, 95% CI = 0.4953–1.127, *p* = .1653) between the two groups of patients with a single tumor diameter <3 cm (Figure 4a,b). However, in patients with a single tumor diameter 3–5 cm, while the difference in OS between the two groups of patients was not statistically significant (HR = 0.6348, 95% CI = 0.245–1.644, *p* = .3514), the RFS was better in SR than in ablation (HR = 0.5392, 95% CI = 0.3057–0.9512, *p* = .0268), as illustrated in Figure 4c,d.

### 3.6 The treatment outcomes of SR and ablation therapy were influenced by the location of the tumor

**FIGURE 5:**
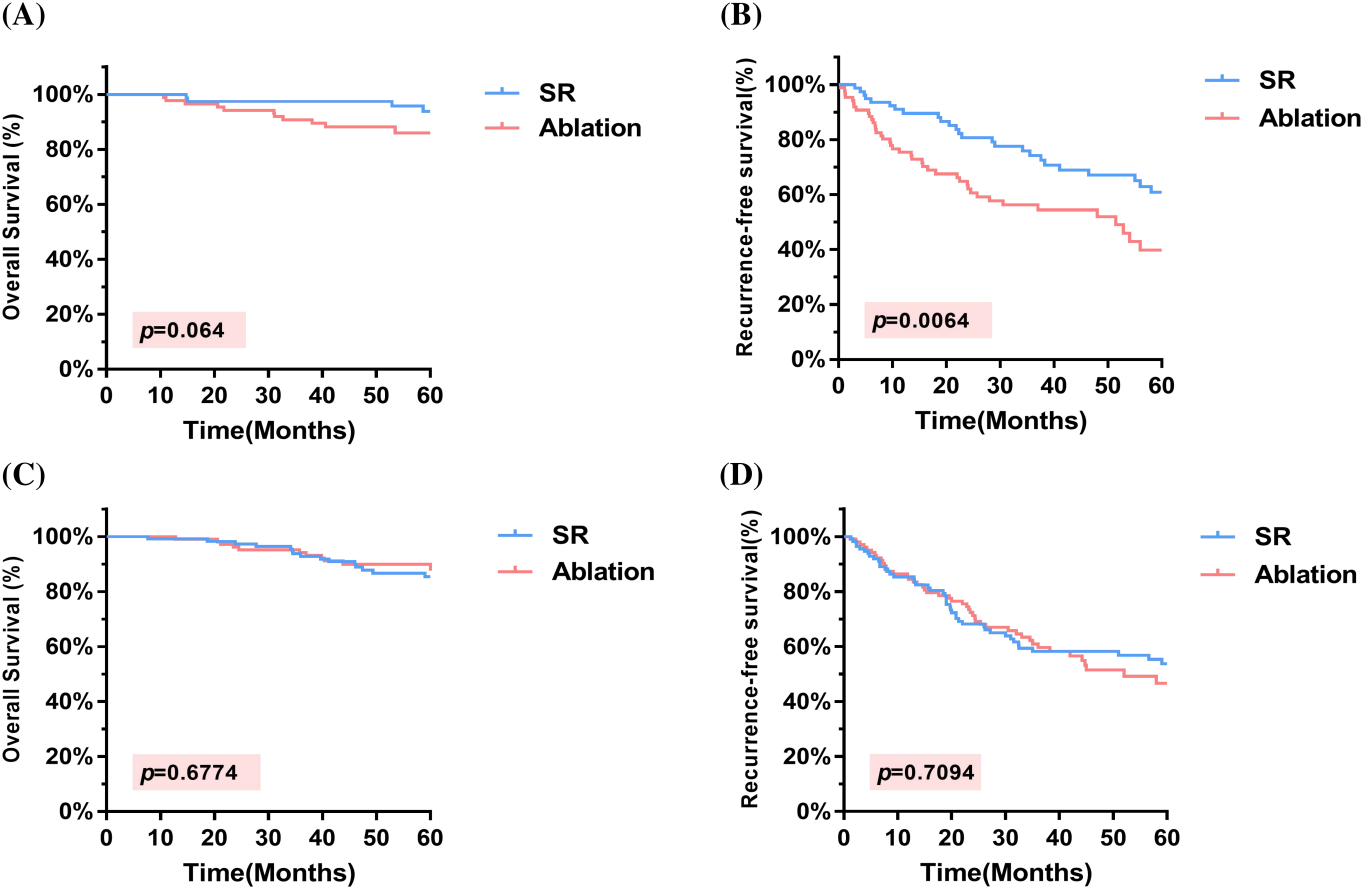
K–M curves comparing OS and RFS in different subgroups. For tumors located in the central region of the liver, there was no statistically significant difference in OS between SR and ablation (A), however, SR was found to be superior to ablation therapy in terms of RFS (B). For tumors located in the noncentral region, there was no significant difference in OS (C) or RFS (D) between the two treatment groups. OS, overall survival; RFS, recurrence‐free survival; SR, surgical resection; HCC, hepatocellular carcinoma.

The location of the tumor was considered when analyzing the treatment outcomes of SR and ablation therapy. Regarding tumors located in the central region (IV, V, VIII) of the liver, there was no statistically significant difference in OS between the SR and ablation groups (*p* = .064). However, SR was superior to ablation therapy in terms of RFS (*p* = .0064). Regarding tumors located in the noncentral region (II, III, VI, and VII), there was no significant difference in OS or RFS between the two treatment groups (*p* = .6774 and *p* = .7094, respectively) (Figure 5). Furthermore, no significant differences in OS or RFS were observed between the SR and ablation groups for tumors located in the left lobe of the liver (*p* = .2319 and *p* = .9208, respectively). However, for tumors located in the right lobe of the liver, both OS and RFS were significantly better with SR than with ablation therapy (*p* = .0212 and *p* = .0072, respectively) (Figure S2).

### 3.7 Risk factors impact the survival and recurrence rate of HCC patients

Cox proportional hazard models were constructed in this study utilizing univariate and multivariate analyses that incorporated various variables, including surgical modality, age, sex, ALBI grade of liver function, tumor diameter, number of tumors, cirrhosis, and alpha‐fetoprotein (AFP) levels. The results from the univariate analysis indicated that HBV infection, a tumor diameter ≥3 cm, an ALBI grade 2 or above, an AFP concentration ≥ 400 ng/mL, and early recurrence significantly impacted OS (*p* < .05). Additionally, multifactorial analysis confirmed that HBV infection, a tumor diameter ≥3 cm, an AFP concentration ≥ 400 ng/mL, and early recurrence were independent risk factors for poor OS (*p* < .05) (Table 3). Moreover, univariate analysis revealed that ablation, a PLT < 100 × 10^9^/L, satellite foci, and the presence of ascites before surgery were statistically significant factors affecting recurrence (*p* < .05). The multifactorial analysis further confirmed that a PLT < 100 × 10^9^/L, an ALBI grade 2 or above, and the presence of satellite foci were independent risk factors for recurrence (*P* < 0.05) (Table 4).

**TABLE 3:** Univariate and multivariate analysis of factors affecting survival (after PSM).

Variables	Univariate			Multivariate		
	HR	95% CI	*p* value	HR	95% CI	*p* value
Surgical procedure (Ablation)	1.269	0.686–2.347	.448			
Age (≥60 years)	1.498	0.793–2.828	.213			
Gender (Female)	0.861	0.338–2.195	.754			
Etiology (HBV)	0.333	0.140–0.793	.013	0.287	0.114–0.718	.008
Liver cirrhosis	1.604	0.766–3.361	.210			
Tumor diameter (≥3 cm)	2.479	1.341–4.582	.004	2.034	1.060–3.902	.033
Number of tumors (2, 3)	0.978	0.302–3.169	.971			
ALBI grade (2–3)	2.029	1.099–3.743	.024	1.329	0.707–2.499	.377
PLT(<100 × 10^9^/L)	1.246	0.636–2.442	.522			
AFP(ng/ml)						
20–400	1.852	0.876–3.914	.107	2.147	0.996–4.627	.051
≥400	2.274	1.037–4.984	.040	2.308	1.043–5.107	.039
Early recurrence	6.929	3.533–13.590	<.001	7.024	3.491–14.133	<.001

Abbreviations: AFP, alpha‐fetoprotein; ALBI grade, albumin‐bilirubin grade; HBV, hepatitis B virus; PLT, platelet; PSM, propensity score matching; SR, surgical resection.

**TABLE 4:** Univariate and multivariate analysis of factors affecting recurrence (after PSM).

Variables	Univariate			Multivariate		
	HR	95% CI	*p* value	HR	95% CI	*p* value
Surgical procedure (Ablation)	1.387	1.015–1.896	.040	1.344	0.912–1.727	.065
Age (≥60 years)	1.274	0.919–1.768	.147			
Gender (Female)	0.785	0.519–1.187	.251			
Liver cirrhosis	1.358	0.958–1.926	.086			
Tumor diameter (≥3 cm)	1.234	0.922–1787	.138			
Number of tumors (2, 3)	1.513	0.889–2575	.127			
ALBI grade (2–3)	1.684	1.232–2.303	.001	1.590	1.153–2.192	.005
PLT(<100 × 10^9^/L)	1.634	1.174–2.274	.004	1.446	1.029–2.033	.034
AFP(ng/ml)						
20–400	1.039	0.723–1.492	.838			
≥400	1.306	0.887–1.923	.176			
Satellite foci	1.847	1.128–3.007	.015	1.810	1.017–2.958	.018

Abbreviations: AFP, alpha‐fetoprotein; ALBI grade, albumin‐bilirubin grade; PLT, platelet.

## 4 DISCUSSION

HCC is the most prevalent primary liver malignancy, and recent advancements in surveillance efforts targeting individuals at high risk of developing HCC have led to an increase in the number of early‐stage HCC diagnoses.^26^ Consequently, the accurate selection and optimization of initial treatment options have gained paramount importance in determining the prognosis of patients with early‐stage HCC.^27^ While previous studies have explored the effectiveness of SR and ablation techniques for treating early‐stage or Milan‐eligible HCC, a consensus has yet to be reached regarding their comparative outcomes. Considering this research gap, the objective of the present study was to conduct a single‐center retrospective cohort study to compare the efficacy of SR and ablation techniques for managing early‐stage HCC.

Our study revealed that, before PSM, compared to the surgical resection group, the ablation group had certain distinguishing characteristics. Specifically, patients in the ablation group were older and exhibited lower white blood cell, platelet, and albumin levels. Conversely, they exhibited higher levels of bilirubin and AST. Additionally, the ablation group had a greater proportion of patients classified as Child–Pugh grade B and having single tumors, albeit smaller in size. These findings suggest that in clinical practice, patients with poorer liver function, compromised systemic conditions, and smaller tumor diameters are more likely to undergo ablation as a treatment modality. Importantly, this treatment choice introduces a potential risk factor for recurrence and survival in patients with HCC, potentially having a substantial impact on the outcomes of our study. Balancing all variables after PSM increased the credibility of the study results.

During the perioperative period, ablation significantly reduced intraoperative bleeding and transfusion rates, and patients who underwent ablation had lower postoperative opioid analgesic use, shorter hospital stays and lower hospital costs than those who underwent resection. In terms of complications, SR patients had a significantly greater incidence of Grade II or above complications than did ablation patients. Although a few patients experienced more severe complications, such as bleeding, biliary leakage, pleural and abdominal effusion, and liver failure, these complications were effectively managed after aggressive treatment involving fluid rehydration and expansion, surgical suturing, and drainage of effusion fluid. These observations not only highlight the less invasive and traumatic nature of ablation but also underscore its superior short‐term recovery outcomes in comparison to those of SR, generally aligning with existing reports.^28^

Lee's prospective study revealed no statistically significant difference in OS between hepatectomy and radiofrequency ablation (RFA) but indicated superior RFS.^23^ Similarly, Hung et al. and Wang et al. yielded the same results.^22^, ^24^ These studies have consistently reported comparable survival rates for SR and RFA in early‐stage HCC, but significantly lower recurrence rates with SR than with RFA. In our study, before and after PSM, patients with early‐stage HCC and those who underwent resection and resection had similar survival outcomes. Nevertheless, patients who underwent SR had a more favorable RFS than those who underwent ablation.

Despite the superior RFS in the SR group compared with the ablation group, there was no difference in the final OS between the two groups. The favorable OS observed in both groups can be attributed to several factors. First, our study exclusively included patients with very early‐stage or early‐stage HCC according to the Barcelona staging system. Second, more than 90% of the enrolled patients had isolated tumors and relatively small tumor diameters. More than half of the patients had tumor diameters less than 2 cm, allowing for better ablation margins, including microsatellite nodules around the tumor.^29^ Additionally, more than 80% of patients had Child–Pugh class A tumors, indicating good liver function, and facilitating effective follow‐up treatments. As mentioned in the study by Huang et al.,^16^ the disparity in RFS can be explained by the variance in tumor clearance achieved through the two treatments. HCC tumor cells primarily disseminate through the portal bloodstream, revealing why HCC frequently spreads within the same liver segment along portal branches.^16^ SR involves the thorough removal of segmental portal vein branches, effectively eliminating potential tumor emboli and the primary tumor within the same segment. Ablation of HCC tissue frequently necessitates multiple electrode insertions and ablations, presenting challenges in achieving fully overlapping ablations under 2D imaging guidance.^16^, ^23^ This may be an important reason for the superior RFS rate of SRs compared with ablations. However, early tumor recurrence did result in increased hospital admissions and salvage treatment. In this study, most patients underwent a combination of treatments, including repeat liver resection, ablation, TACE, targeted therapy and immunotherapy with PD‐1/PD‐L1 inhibitors, more frequently after tumor recurrence, all of which may have affected the final survival outcomes observed in our study.

The diameter and number of tumors are crucial to the choice of clinical treatment, and treatment guidelines offer different recommendations based on these factors. Local ablation has been reported to be highly effective for patients with a tumor diameter less than 3 cm.^30^ Accordingly, we categorized our patients into two subgroups: those with single tumors measuring less than 3 cm in diameter and those with single tumors ranging from 3 to 5 cm in diameter. Our study showed that patients with single tumors measuring less than 3 cm had similar OS and RFS rates with SR and ablation, which is in line with the majority of previous studies.^10^, ^11^, ^12^ Conversely, among patients with single tumors ranging from 3 to 5 cm in diameter, our results demonstrated that OS was comparable in both groups, but SR was significantly better than ablation in terms of RFS, which is generally consistent with the results of a retrospective study by Wang et al.^31^ Notably, Wang et al.'s study revealed comparable DFS between the two treatment groups when compared to patients enrolled in later years, indicating that ablation could serve as a potential alternative to SR for 3–5 cm tumors when the technology matures. Zheng et al. reported that SR and ablation provided similar outcomes for patients with 3–5 cm long HCC lesions, but their study had a limited sample size.^32^ Therefore, further studies with larger sample sizes are needed regarding the treatment of 3–5 cm long HCC lesions.

The impact of tumor location on postoperative survival outcome in patients with HCC has attracted widespread attention in recent years, yet a consensus regarding treatment remains elusive. Based on the Couinaud classification of the liver, tumors located in segments IV, V and VIII of the liver are classified as the central type of HCC.^33^ These tumors possess a unique anatomical location and present considerable surgical challenges due to their proximity to vital liver vessels, such as the portal vein, hepatic vein, and inferior vena cava. Historically, SRs of central HCC patients have engendered serious complications, such as hemorrhage and liver failure, due to massive resection of the liver parenchyma, but the incidence of these serious complications has been substantially mitigated in recent years due to advancements in surgical techniques, approaches, and instruments. Orimo et al. conducted an analysis comparing the efficacy of central hepatectomy and major hepatectomy for central HCC and reported no significant difference in short‐ or long‐term survival rates or recurrence between the two approaches.^34^ Similarly, a meta‐analysis yielded the same outcome,^35^ with no difference in complications between the two treatments. Overall, the SR of central HCC was generally considered safe and dependable. A review of the literature revealed limited studies comparing the effectiveness of SR and ablation for central HCC.

Thus, in this study, we undertook a separate comparison of SR and ablation for central and noncentral HCC patients to assess their respective efficacies. The results showed that there was no significant difference in OS between SR and ablation for central HCC, while SR was associated with a lower recurrence rate than ablation. However, in noncentral HCC patients, there was no difference in OS or RFS between the two groups. The disparity in outcomes can be attributed to several factors. First, HCC located in the central region is challenging due to its deep location and difficulty in localization. Percutaneous ablation requires caution to avoid damage to adjacent blood vessels, potentially resulting in incomplete ablation. Moreover, the proximity of the tumor to large blood vessels can cause a heat sink effect, substantially diminishing the effectiveness of coagulative necrosis and thereby impacting the outcome.^36^ In addition, patients with lesions located in the right lobe of the liver exhibited longer long‐term OS and RFS when treated with SR than when treated with ablation. The segmental distribution of HCC has been previously reported to be proportional to the volume of the liver lobe or other lobes.^37^ In this study, a high incidence of HCC was observed in the right lobe of the liver (74.3%), mainly in hepatic segments V, VII and VIII, consistent with the findings reported by Renzulli et al.^38^ Tumor location in segment VIII, which is proximal to the diaphragm, was identified as an important factor influencing the effectiveness of ablation, and location in this segment was associated with a 3.5‐fold greater risk of microvascular invasion (MVI) than location in other liver segments. Notably, local recurrence of HCC in segments VII and VIII occurred after ablation. The proximity to the diaphragm was an independent predictor of local recurrence after ablation, as positioning the ablation probe was more difficult.^39^

The recurrence rate of HCC following SR remains high, significantly impacting patient survival and prognosis. Studies have reported a recurrence rate of 50–70% at 5 years postsurgery.^40^ Addressing postoperative recurrence and improving survival have become prominent research foci. Numerous studies have demonstrated several high‐risk factors for postoperative recurrence and survival, including large tumor size, multiple tumors or satellite foci, tumor envelope invasion or absence, MVI, and high AFP levels.^41^ In our study, HBV infection, tumor diameter ≥3 cm, early recurrence, and AFP≥400 ng/mL were identified as independent risk for low OS; PLT < 100 × 10^9^/L, ALBI grade 2 or above, and satellite foci were independent risk factors for recurrence. Surgery‐induced immunosuppression has been associated with an increased risk of HBV reactivation, leading to cirrhosis or the accelerated progression of preexisting cirrhosis and ultimately resulting in HCC recurrence. Routine postoperative treatment with antiviral drugs effectively inhibits HBV reactivation and reduces the level of inflammation in the residual liver, delaying the progression of cirrhosis or liver failure to some extent. Tumor size not only correlates with invasive and metastatic potential but also affects the difficulty of treatment. Larger tumors pose challenges during surgery, increase postoperative liver burden, and increase the risk of liver failure. In ablation approaches, a larger tumor diameter requires repeated insertion and ablation, making it difficult to accurately cover the entire liver area in three dimensions under the guidance of two‐dimensional ultrasound and possibly leading to incomplete ablation or the risk of needle tract metastasis,^17^ which favor a poor prognosis. AFP is a widely used serum tumor indicator in clinical HCC diagnosis,^42^ and previous studies have confirmed that AFP can promote cancer cell proliferation, motility, invasive growth, and metastasis in various HCC cell lines or animal models.^43^ In this study, a preoperative AFP concentration ≥ 400 ng/mL was identified as a risk factor for shorter OS after surgery, although no statistically significant differences were observed in terms of recurrence. Liver function status represents an important preoperative assessment in HCC patients and is closely related to the occurrence of liver failure during the perioperative period and the postoperative prognosis. The ALBI grade is an index used to evaluate liver reserve function, with a higher ALBI grade representing poorer liver function. A study revealed that the ALBI grade was strongly correlated with patient prognosis and had a greater predictive value than the Child–Pugh grade.^44^ Therefore, we included the ALBI grade in the Cox regression analysis, and a high ALBI grade was found to be an independent risk factor for both survival and recurrence. Platelets are used mainly for hemostasis after vascular injury, and in recent years, some studies have demonstrated that platelets may play a role in the hematogenous metastasis of HCC. With respect to the prognosis after hepatectomy, some researchers have found that thrombocytopenia is a risk factor for the recurrence of HCC after surgery.^45^ Our study obtained similar results, although the exact mechanism remains unknown. Platelets may promote liver regeneration and the growth of HCC cells, warranting further investigation. Satellite foci around tumor nodes in HCC are caused mainly by intrahepatic metastases, reflecting the aggressiveness of the tumor. In the present study, satellite foci were identified as an independent risk factor for the recurrence of HCC after surgery, consistent with the findings of recent studies and expert consensus. Donat et al. also reported 5‐year recurrence rates of 37.5% and 16.8% for HCC patients with and without satellite foci, respectively.^46^

This study has inherent limitations. As a single‐center study, this study inevitably introduced potential selection and indication biases. Given that the study was retrospective, there may have been loss to follow‐up during the follow‐up process. Despite some minor limitations, this clinical study has notable strengths, rendering it valuable. In recognition of its single‐center retrospective cohort design, PSM was implemented to alleviate the impact of confounding and selection bias. Furthermore, we compared treatment outcomes for patients with tumors of varying diameters and locations and followed up for a longer period of time, contributing valuable insights to guide clinical treatment decisions.

## 5 CONCLUSION

In conclusion, this study serves as a valuable foundation for the application of ablation in the management of early‐stage HCC. The minimally invasive nature of ablation provides important advantages, positioning it for wider application in the treatment of HCC patients with a tumor size less than 3 cm. However, it is worth noting that over 90% of the patients included in this study had isolated tumors. Thus, further research and analysis are warranted to ascertain whether the results are the same for patients with multiple small HCC lesions.

## AUTHOR CONTRIBUTIONS

**Bilan Yang:** Data curation (equal); formal analysis (equal); writing – original draft (equal). **Xiaoli Xi:** Data curation (equal); formal analysis (equal); writing – original draft (equal). **Hongsheng Yu:** Data curation (equal); validation (equal). **Hao Jiang:** Data curation (equal); validation (equal). **Zixi Liang:** Data curation (equal); validation (equal). **Abdukyamu Smayi:** Data curation (equal); validation (equal). **Bin Wu:** Funding acquisition (equal); supervision (equal); validation (equal); writing – review and editing (equal). **Yidong Yang:** Funding acquisition (equal); supervision (equal); validation (equal); writing – review and editing (equal).

## FUNDING INFORMATION

This work was supported by grants from the Natural Science Foundation of Guangdong Province for Distinguished Young Scholar (2022B1515020024), the National Natural Science Foundation of China (82070574), the Natural Science Foundation Team Project of Guangdong Province (2018B030312009).

## CONFLICT OF INTEREST STATEMENT

The authors declare no conflict of interest.

## ETHICS STATEMENT

Study protocols were approved by the Ethics Committee of the Third Affiliated Hospital of Sun Yat‐Sen University ([2019]02–530‐01). Informed consent was obtained from all the participants included in this study according to the committee regulations.

## Supporting information


**Figure S1.** Total population of HCC by age and sex.
**Figure S2.** K–M curves comparing OS and RFS in different subgroups. For tumors located in the left lobe of the liver, there was no significant difference in OS (a) or RFS (b) between the SR and ablation groups. For tumors located in the right lobe of the liver, both OS (c) and RFS (d) were significantly better with SR than with ablation therapy. OS, overall survival; RFS, recurrence‐free survival; SR, surgical resection; HCC, hepatocellular carcinoma.

**Table S1.** General clinical information of patients in SR and ablation (after PSM).
**Table S2.** Postoperative complications in SR and ablation (after PSM).
**Table S3.** First recurrence and treatments in the SR and ablation groups.

## Data Availability

The data that support the findings of this study are available on request from the corresponding author.
