# Supplementary material for: Evaluation of the effectiveness of surgical resection and ablation for the treatment of early‐stage hepatocellular carcinoma: A retrospective cohort study
Source: Cancer Rep (Hoboken). 2024 Mar 15;7(3):e2030. doi: 10.1002/cnr2.2030 (PMC10941592; doi:10.1002/cnr2.2030)
Supplement: Supplementary file 1 — Figure S1. Total population of HCC by age and sex. Figure S2. K–M curves comparing OS and RFS in different subgroups. For tumors located in the left lobe of the liver, there was no significant difference in OS (a) or RFS (b) between the SR and ablation groups. For tumors located in the right lobe of the liver, both OS (c) and RFS (d) were significantly better with SR than with ablation therapy. OS, overall survival; RFS, recurrence‐free survival; SR, surgical resection; HCC, hepatocellular carcinoma. [file CNR2-7-e2030-s001.pptx]

## Slide 1
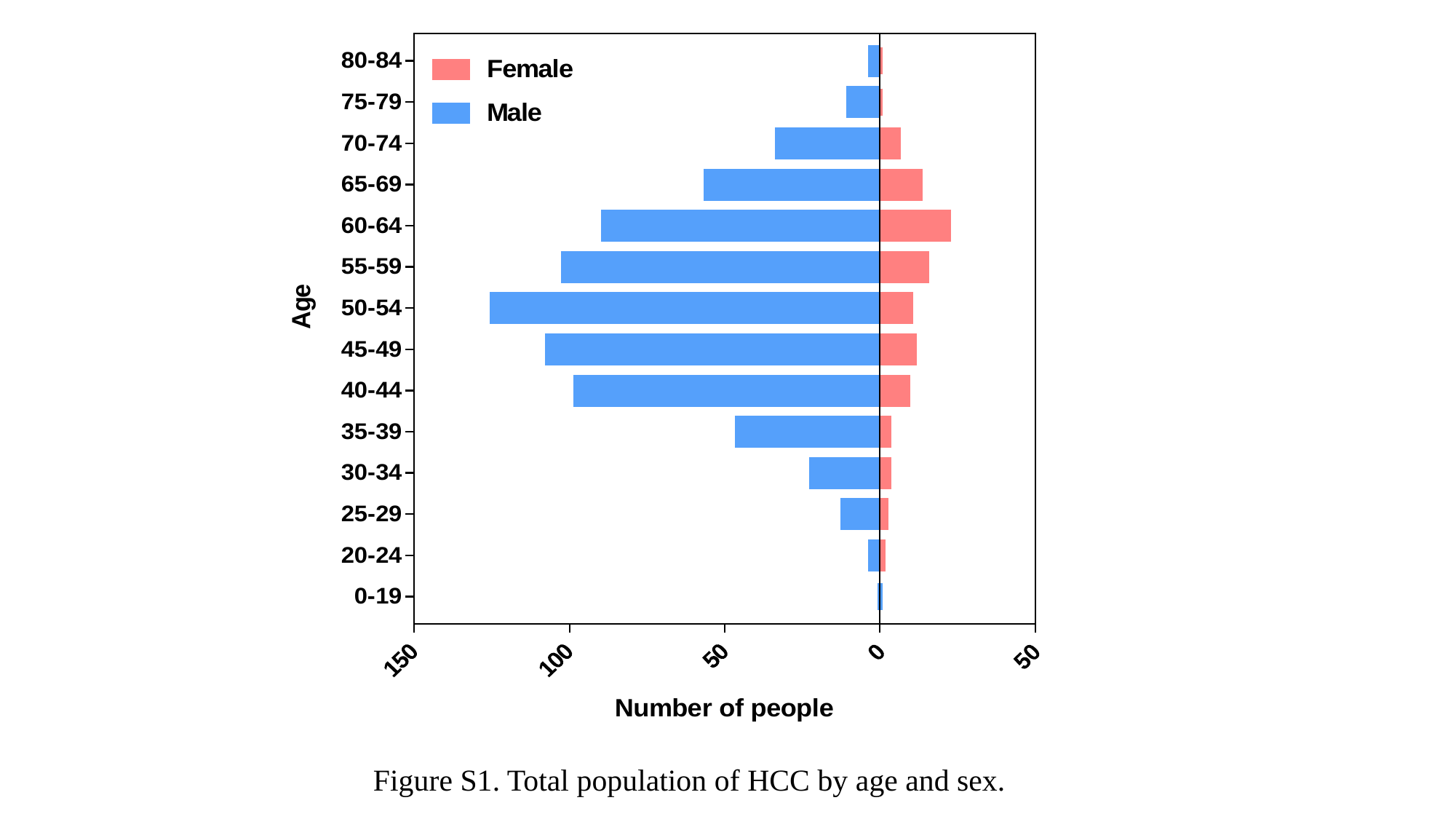

Figure S1. Total population of HCC by age and sex.

## Slide 2
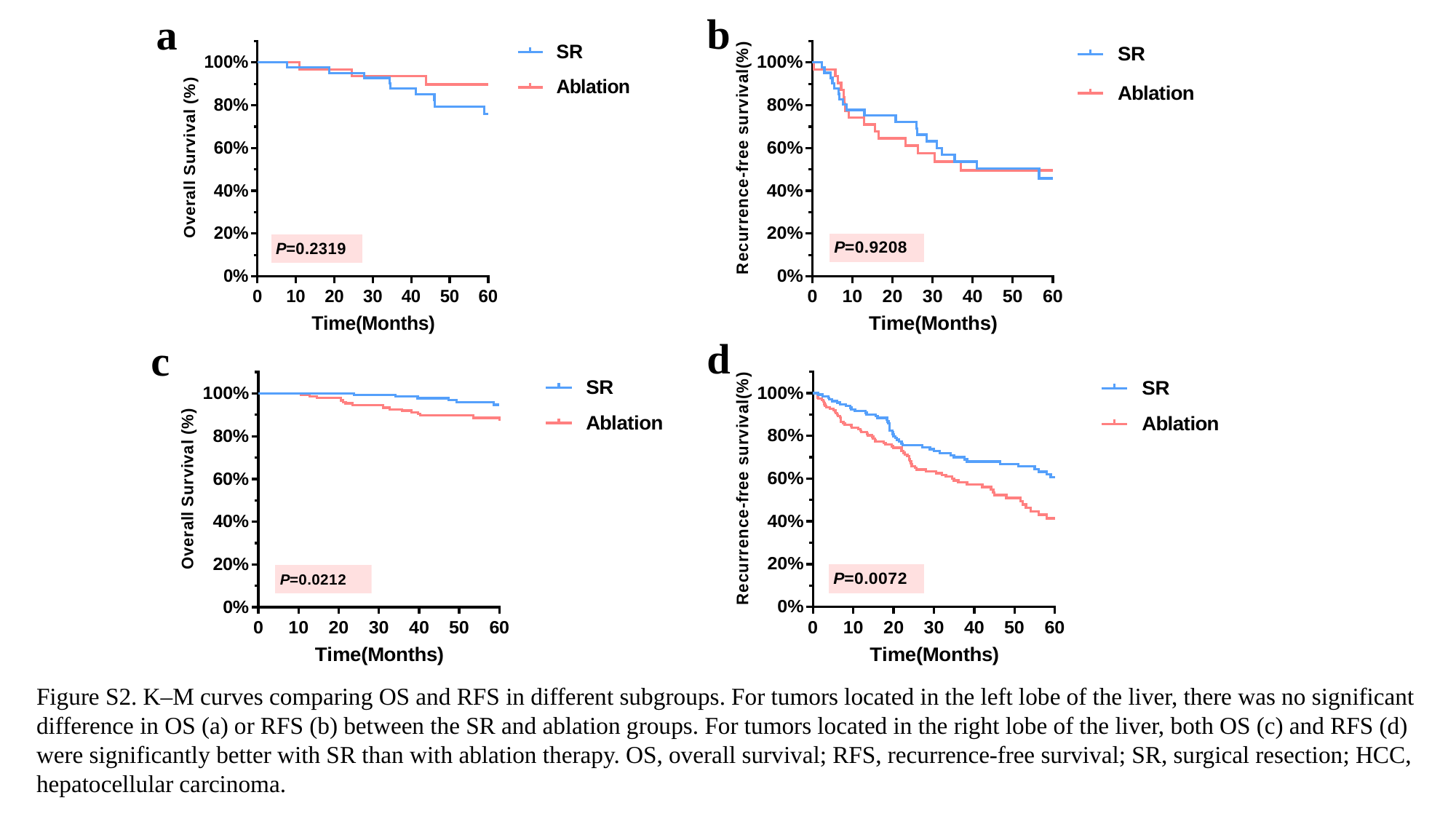

b
a
d
c
Figure S2. K‒M curves comparing OS and RFS in different subgroups. For tumors located in the left lobe of the liver, there was no significant difference in OS (a) or RFS (b) between the SR and ablation groups. For tumors located in the right lobe of the liver, both OS (c) and RFS (d) were significantly better with SR than with ablation therapy. OS, overall survival; RFS, recurrence-free survival; SR, surgical resection; HCC, hepatocellular carcinoma.
