# Supplementary material for: Evaluation of the effectiveness of surgical resection and ablation for the treatment of early‐stage hepatocellular carcinoma: A retrospective cohort study
Source: Cancer Rep (Hoboken). 2024 Mar 15;7(3):e2030. doi: 10.1002/cnr2.2030 (PMC10941592; doi:10.1002/cnr2.2030)
Supplement: Supplementary file 2 — Table S1. General clinical information of patients in SR and ablation (after PSM). Table S2. Postoperative complications in SR and ablation (after PSM). Table S3. First recurrence and treatments in the SR and ablation groups. [file CNR2-7-e2030-s002.docx]

Table S1 General clinical information of patients in SR and ablation (after PSM)

| Variables | SR (n=189) | Ablation (n=189) | *P* value |
| --- | --- | --- | --- |
| Age (Year ) | 53.4±10.6 | 52.9±11.4 | *P*=0.692 |
| Sex (n,%) |  |  | *P*=0.881 |
| Male | 164(86.8) | 162(85.7) |  |
| Female | 25(13.2) | 27(14.3) |  |
| Viral infections (n,%) |  |  |  |
| HBV | 176(93.1) | 178(93.2) | *P*=0.833 |
| HCV | 4(2.1) | 4(2.1) | *P*=1.000 |
| Cirrhosis (n,%) | 131(69.3) | 130(68.8) | *P*=1.000 |
| Family History (n,%) | 15(7.9) | 16(8.5) | *P*=1.000 |
| Combine chronic diseases (n,%) | 39(20.6) | 36(19.0) | *P*=0.797 |
| BMI | 22.8±3.0 | 22.9±3.0 | *P*=0.708 |
| WBC (×10^9^/L) | 5.4(2.4) | 5.4(2.2) | *P*=0.893 |
| Hb (g/L) | 141(20) | 141(23) | *P*=0.847 |
| PLT (×10^9^/L) | 145(98) | 142(86) | *P*=0.659 |
| AST (U/L) | 33(20) | 33(23) | *P*=0.951 |
| ALT (U/L) | 31(28) | 35(27) | *P*=0.252 |
| ALB (g/L) | 41(5.9) | 40.9(5.3) | *P*=0.648 |
| TBil (umol/L) | 14(8.3) | 13(8.6) | *P*=0.435 |
| AFP (ng/ml) | 38.9(359.8) | 22.3(216.5) | *P*=0.274 |
| Child-Pugh classification (n,%) |  |  | *P*=0.608 |
| A | 168(88.9) | 172(91.0) |  |
| B | 21(11.1) | 17(9.0) |  |
| Number of tumours (n,%) |  |  | *P*=0.845 |
| Single | 176(93.1) | 174(92.1) |  |
| Multiple | 13(6.9) | 15(7.9) |  |
| Tumour diameter (mm) | 25(12) | 24(12) | *P*=0.579 |

**Abbreviations**: SR, surgical resection; PSM, propensity score matching; HBV, hepatitis B virus; HCV, hepatitis C virus; BMI, Body Mass Index; Hb, hemoglobin; PLT, platelet; ALT, alanine aminotransferase; AST, aspartate transaminase; ALB, [albumin](javascript:;); TBil, total bilirubin; AFP, alpha-fetoprotein.

Table S2 Postoperative complications in SR and ablation (after PSM)

| Variables | SR  n=189 | Ablation  n=189 | *P* value |
| --- | --- | --- | --- |
| Dindo-Demartines-Clavien complication classification (n,%) |  |  |  |
| I | 42(22.2) | 44(23.2) | - |
| II | 10(5.3) | 7(3.7) | - |
| III | 13(6.9) | 4(2.1) | - |
| IV | 5(2.6) | 1(0.5) | - |
| Grade II or above complications (n,%) | 28(14.8) | 12(6.3) | ***P*=0.011** |

**Abbreviations**: SR, surgical resection; PSM, propensity score matching.

Table S3 First recurrence and treatments in the SR and ablation groups

| Variables | SR  n=70 | Ablation  n=87 |
| --- | --- | --- |
| Site of recurrence, n (%) |  |  |
| Liver | 66(94.3) | 82(94.3) |
| Extrahepatic | 4(5.7) | 2(2.3) |
| Bone or lymph node or others | 0(0) | 3(3.4) |
| Treatments for first recurrence, n (%) |  |  |
| Surgical resection | 12(17.1) | 15(17.2) |
| Ablation | 34(48.6) | 45(51.7) |
| SR and Ablation | 2(2.9) | 2(2.3) |
| TACE | 12(17.1) | 13(14.9) |
| Targeted therapy/immunotherapy | 0(0) | 2(2.3) |
| Radiotherapy | 0(0) | 1(1.1) |
| Liver transplantation | 1(1.4) | 0(0) |

**Abbreviations**: SR, surgical resection; PSM, propensity score matching; TACE, transcatheter arterial chemoembolization
